# “SPLIT” Pancreaticojejunostomy in the Surgical Treatment of Chronic Pancreatitis

**DOI:** 10.1155/1994/56303

**Published:** 1994

**Authors:** W. Mulder, E. de Jong, T. M. van Gulik, L. Th. de Wit, D. J. van Leeuwen, P. C. M. Verbeek, M. N. van der Heyde

**Affiliations:** Academic Medical Center University of Amsterdam Department of Surgery Meibergdreef 9 Amsterdam 1105 AZ The Netherlands

## Abstract

“Split” pancreaticojejunostomy is a procedure consisting of vertical transection of the pancreas and anastomosis of both sides of the cut pancreatic duct with an interposed, Roux-en-Y jejunal loop. In this paper we report the long term results of this procedure in the treatment of eight patients with chronic pancreatitis (CP).


*HPB Surgery*, 1994, Vol. 8, pp. 9–12
Reprints available directly from the publisher
Photocopying permitted by license only

© 1994 Harwood Academic Publishers GmbH
Printed in Malaysia

### "SPLIT" Pancreaticojejunostomy in the Surgical Treatment of Chronic Pancreatitis

W. MULDER, E. DE JONG, T. M. VAN GULIK, L. TH. DE WIT, D. J. VAN LEEUWEN, P. C. M. VERBEEK and M. N. VAN DER HEYDE

Academic Medical Center, University of Amsterdam, Department of Surgery, Meibergdreef 9, 1105 AZ Amsterdam, The Netherlands

"Split" pancreaticojejunostomy is a procedure consisting of vertical transection of the pancreas and anastomosis of both sides of the cut pancreatic duct with an interposed, Roux-en-Y jejunal loop. In this paper we report the long term results of this procedure in the treatment of eight patients with chronic pancreatitis (CP).

KEY WORDS: Chronic pancreatitis pancreaticojejunostomy.

### INTRODUCTION

The main indication for surgical treatment of chronic pancreatitis (CP) is intractable pain. Since an increase of intraductal pressure has been incriminated with respect to the origin of pancreatic pain[1,2], a number of operative procedures have been devised aiming at relief of intraductal pressure by drainage of the pancreatic duct. The most common of these drainage procedures consist of a distal end-to-end (DuVal[3]) or lateral side-to-side (Puestow[4]) pancreaticojejunostomy. Prerequisite for the successful application of such a procedure, is a readily identifiable dilated pancreatic duct. In 1967 Marvin James described a "split" pancreaticojejunostomy. Following vertical, partial transection of the pancreas he anastomosed both ends of the pancreatic duct to the sides of a closed Roux-en-Y limb of jejunum, or he performed an onlay pancreaticojejunostomy, suturing the open end of the Roux-en-Y limb to the edges of the partially transected pancreas[5].

We have applied a modified version of this method in eight patients with CP, in which the pancreatic duct was not obviously dilated.

### PATIENTS AND METHODS

The operative methods of "split" pancreaticojejunostomy consisted of total transection of the pancreas in the region of the corpus allowing prompt identification of the sectioned pancreatic duct. In most cases the pancreatic duct was less than 3 mm in diameter. Both sides of the duct were anastomosed face to face, to a Roux-en-Y jejunal loop using a mucosa-to-mucosa technic (Figure 2). Double, transanastomotic silicon stents were left for ten days. The anastomoses were visualised radiographically by injection of gastrografine through the stents before removal.

From 1986 until 1988 we performed this "split" pancreaticojejunostomy in eight patients (five male, three female), suffering from severe upper abdominal pain caused by CP (Table 1). The mean age at the time of operation was 35 years (range 13–53 years). Four patients were diagnosed as having an alcohol induced CP, three suffered from a familial pancreatitis having several members of their family suffering from non-alcohol related CP and in one patient, CP was considered to be idiopathic, since no indication of familiar incidence, alcohol abuse or congenital pancreatic malformations could be identified.

Two of the patients had undergone previous marsupialisation of a pseudocyst. ERCP in all patients showed irregular but non-dilated main pancreatic ducts, precluding the use of a lateral side-to-side pancreaticojejunostomy (Figure 1). Clinical outcome was classified as "good" if the patient was almost or totally painfree without the need of any analgetics, "fair" if the pain had improved and only occasionally required

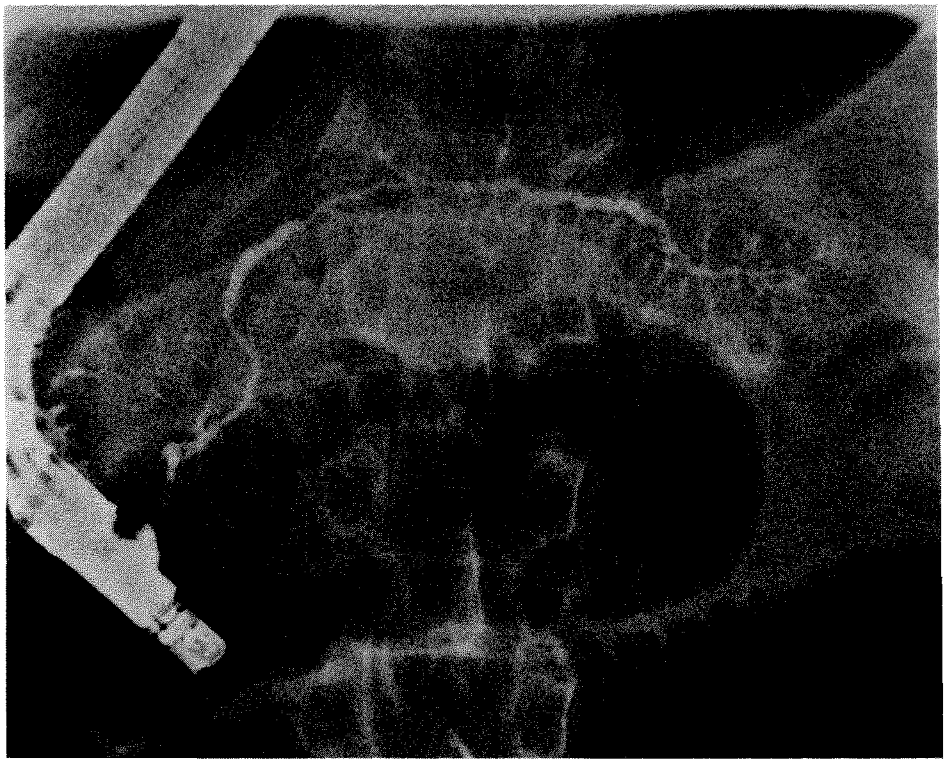

**Figure 1** Preoperative ERCP showing an irregular, non-dilated main pancreatic duct.

**Table 1** Patients and results.

| *Male/female* | *Age (years)* | *Etiology* | *Postop. compl.* | *Treatment* | *Follow-up (months)* | *Result* |
|---|---|---|---|---|---|---|
| 1. M | 53 | alcohol induced | abscess | percutaneous drainage | 20 | good |
| 2. M | 36 | alcohol induced | — | — | 44 | bad |
| 3. M | 44 | alcohol induced | ARDS sepsis | mult.*) relap. | 50 | fair |
| 4. M | 36 | alcohol induced | — | — | 49 | bad |
| 5. F | 36 | idiopathic | — | — | 43 | bad |
| 6. F | 13 | familial | — | — | 20 | good |
| 7. F | 25 | familial | — | — | 34 | good |
| 8. M | 39 | familial | — | — | 31 | good |

*) complications were due to a leaking cystoduodenostomy which was performed at the same time.

non-morphine type analgetics, and "bad" if there was no improvement at all.

### RESULTS

All patients were assessed on an out-patient basis during a follow-up period ranging from 20 to 50 months. Four patients had a good result, one fair, and three had a bad result (Table 1).

None of the patients had any disturbance of endocrine function preoperatively, nor postoperatively.

Exocrine pancreatic dysfunction causing diarrhea was present preoperatively in two patients. One of them showed no amelioration after operation and still

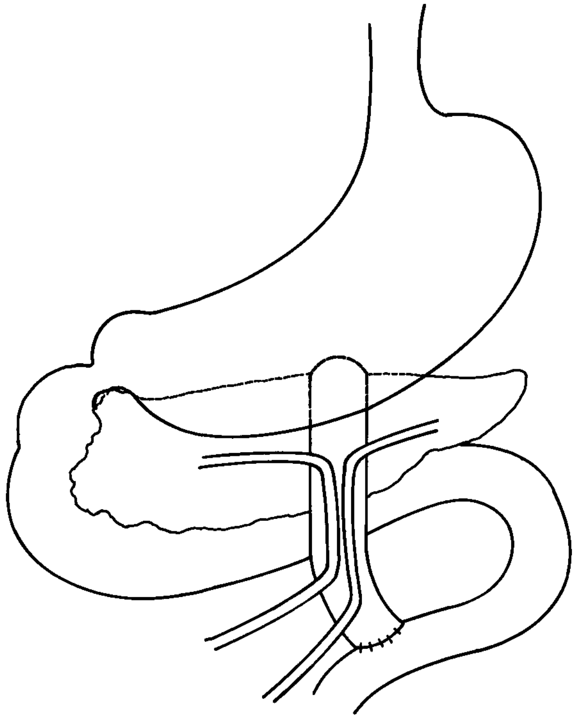

**Figure 2** Operative technique of "split" pancreaticojejunostomy. Transection of the pancreas is undertaken in the region of the corpus. After identification of the sectioned pancreatic duct, both sides are anastomosed to a Roux-en-Y jejunal loop.

needed pancreatic enzyme supplementation. The second patient only had a short period of diarrhea postoperatively, that responded well to medication.

Postoperative complications consisted of an intraabdominal abscess in one patient and an episode of sepsis and ARDS in conjunction with a pancreaticocystoduodenostomy in another patient.

### DISCUSSION

Objective assessments to quantitate chronic pancreatitis are lacking to this day. Both endocrine and exocrine function are not necessarily impaired by CP. The clinical impact of the disease is, most importantly, determined by the pain as experienced by the patient. Therefore, we chose to evaluate our results primarily by regarding relief of pain in our patients. The classification method used in this study is simple and turned out to be very practicable. Most patients were doing either very well or badly.

The patients that did not do well at follow-up had problems caused mainly by persistence of their drinking habit. These patients constitute a well known problem in the assessment of any treatment of chronic pancreatitis[6]. Only one patient with a history of alcohol abuse was doing well after operation (follow-up 20 months). In particular, the patients with familial pancreatitis all had good results after "split" pancreaticojejunostomy.

A non-dilated pancreatic duct was no impairment to carrying out this technique, since the duct could be readily identified in the cut-surface of the pancreas.

Apart from one intraabdominal abscess, the method was not associated with serious complications. The patient who had developed sepsis and ARDS, was shown to have leaked from a concomitant drainage of a pancreatic pseudocyst.

In conclusion, we consider "split" pancreatico jejunostomy as an option in the operative treatment of chronic pancreatitis of the familial type and of alcoholic origin when a conventional, lateral pancreaticojejunostomy is not feasible.

### REFERENCES

1. Bradley, E. L. (1982) Pancreatic duct pressure in chronic pancreatitis.*Am. J. of Surg.*, **144**, 313–316.
2. Cahow, E., and Hayes, B. A. (1973) Operative treatment of chronic recurrent pancreatitis. *Am. J. of Surg.*, **12**, 390–398.
3. DuVal, M. K. (1954) Caudal pancreaticojejunostomy for chronic relapsing pancreatitis. *Ann. Surg.*, **144**, 775.
4. Puestow, C. B., and Gillespy, W. J. (1958) Retrograde surgical drainage of pancreas for chronic relapsing pancreatitis. *Arch. Surg.*, **76**, 898.
5. James, M. (1967) Treatment of pancreatic duct obstruction by "split" pancreaticojejunostomy. *The American Surgeon*, **33**, 1–6.
6. Worning, H. Chronic pancreatitis–epidemiology, etiology and clinical picture. 1946–1984. 1984 Pancreatitis. Concepts and classification: 347–350.
7. Catell, R. B. (1947) Anastomosis of the duct of Wirsung. *Surg. Clin. North. Am.*, **27**, 636.
8. Durbec, J. P., and Sarles, H. Epidemiology of chronic pancreatitis. 1984 Pancreatitis. Concepts and classification: 371–376.
9. Frey, C. F., Child, C. G., and Fry, W. (1976) Pancreatectomy for chronic pancreatitis. *Ann. Surg.*, **184**, 403–414.
10. Fry, W. J., and Child, C. G. (1965) Ninety-five percent distal pancreatectomy for chronic pancreatitis. *Ann. Surg.*, **162**, 543.
11. Leger, L., Lenriot, J. P., and Lemaigre, G. (1974) Five to twenty years follow-up after surgery for chronic pancreatitis in 148 patients. *Ann. Surg.*, **180**, 185–191.
12. Pain, J. A., and Knight, M. J. (1988) Pancreaticogastrostomy: the preferred operation for pain relief in chronic pancreatitis. *Br. J. of Surg.* **75**, 220–222.
13. Partington, P. F., and Rochelle, REL. (1960) Modified Puestow procedure for retrograde drainage of the pancreatic duct. *Ann. Surg.*, **152**, 1037.
14. Sarles, H. (1986) Chronic pancreatitis: Etiology and pathophysiology. The exocrine pancreas: Biology, pathobiology and diseases. V. L. W. Go *et al.* (ed.) 527–540.
15. Thal, A. P. (1962) A technique for drainage of the obstructed pancreatic duct. *Surg.*, **51**, 313.

### INVITED COMMENTARY

The authors have described their experience in the management of a difficult group of patients whom the surgeon may be called on to treat. The technical aspects of their operation are not simple in that the ducts are small and the anastomoses have to be placed precisely. The problem is to place the openings on each side of the jejunum at exactly the right place in its circumference so as to permit tension-free anastomoses.

Identification of the normal sized pancreatic duct is usually achieved fairly readily in the patient with chronic pancreatitis, more easily than in the patient with a normal pancreas. After transecting the pancreas, if the duct cannot be readily identified on the patient's left side, the intravenous injection of secretin, one unit per kilogram of body weight, characteristically results in a flow of pancreatic juice that permits ready identification of the duct. Secretin does not necessarily result in a retrograde flow of juice from the severed right side, however. On one occasion, we have used intraoperative ERCP in which the injected medium included methylene blue dye to identify the opposite side (patient's right side) of the severed duct.

The authors performed the operation in eight patients without technical mishap. This is not an simple operation, however, and anastomotic leakage is certainly to be anticipated should the operation be undertaken by less experienced surgeons. Space has not permitted the authors to describe their operation in sufficient detail to provide technical guidance.

Pancreaticojejunostomy, adapted to the forward flow of pancreatic juice, has been repeatedly demonstrated as a valid technical option for use with small pancreatic ducts. Many such anastomoses have been demonstrated to remain open for prolonged periods. The long term patency, however, of retrograde anastomoses, as illustrated by the authors' anastomoses of the duct on the patient's right side to the jejunum, is not a proven option in the patient *without* obstruction to forward flow. The Duval operation of anastomosis of the duct in the tail of the pancreas to the jejunum, to permit retrograde flow, has been largely abandoned. Continued retrograde flow is probably not a valid option in the absence of obstruction to antegrade flow. Without follow-up endoscopic retrograde pancreatograms done six months to one year after operation, there is little evidence that the right sided anastomoses remained patent.

The results of operations for familial pancreatitis are known to be superior to those for chronic alcoholic pancreatitis. The results might have been as good in both groups, and the operative danger less, had the surgeons oversewn the transected pancreas on the patient's right side, while anastomosing the duct from the patient's left side.

The authors have described an option which should be in the armamentarium of the experienced pancreatic surgeon. The results, however, suggest that its application to the treatment of chronic pancreatitis should be evaluated further before it is accepted for widespread use.

John M. Howard, M. D.
2121 Hughes Drive, Suite # 940
Toledo, OH 43606, United States
(419) 479-2626

